# Electro-thermal control of aluminum-doped zinc oxide/vanadium dioxide multilayered thin films for smart-device applications

**DOI:** 10.1038/srep21040

**Published:** 2016-02-17

**Authors:** J. R. Skuza, D. W. Scott, R. M. Mundle, A. K. Pradhan

**Affiliations:** 1 Center for Materials Research, Norfolk State University, Norfolk, VA, 23504, USA; 2 Department of Engineering, Norfolk State University, Norfolk, VA, 23504, USA

## Abstract

We demonstrate the electro-thermal control of aluminum-doped zinc oxide (Al:ZnO) /vanadium dioxide (VO_2_) multilayered thin films, where the application of a small electric field enables precise control of the applied heat to the VO_2_ thin film to induce its semiconductor-metal transition (SMT). The transparent conducting oxide nature of the top Al:ZnO film can be tuned to facilitate the fine control of the SMT of the VO_2_ thin film and its associated properties. In addition, the Al:ZnO film provides a capping layer to the VO_2_ thin film, which inhibits oxidation to a more energetically favorable and stable V_2_O_5_ phase. It also decreases the SMT of the VO_2_ thin film by approximately 5–10 °C because of an additional stress induced on the VO_2_ thin film and/or an alteration of the oxygen vacancy concentration in the VO_2_ thin film. These results have significant impacts on technological applications for both passive and active devices by exploiting this near-room-temperature SMT.

Vanadium dioxide (VO_2_) in thin-film and nanostructured forms has been intensely studied in recent years because of the presence of a metal-insulator (MIT) or semiconductor-metal transition (SMT) near room temperature (RT) at ~68 °C in bulk form, which is also accompanied by a structural phase transition^1^,^2^,^3^,^4^,^5^,^6^,^7^. The crystal symmetry of VO_2_ changes from a *P*2_1_/*c* (monoclinic semiconducting phase) to a *P*4_2_/*mnm* (rutile metallic phase) space symmetry when VO_2_ traverses from below to above the SMT. This transition can be temperature-1, voltage-8,9,10, and photo-induced^11^ in this strongly electron-correlated material. The successful growth and study of VO_2_ thin films has been demonstrated using several deposition techniques, including reactive sputtering^12^, atomic layer deposition (ALD)^13^, pulsed laser deposition (PLD)^14^, chemical vapor deposition^15^, electron beam evaporation^16^, the sol-gel process^17^, and thermal evaporation^18^. These and many other studies have provided a better understanding of the SMT and VO_2_ properties; the oxygen vacancy concentration^19^ and strain^20^ play important roles, but many aspects remain unclear. A more thorough understanding of the SMT and VO_2_ properties will aid in the fine-tuning and control of the SMT so that it can be more reliably used for applications.

Technological applications in various passive and active devices have been developed based on this near-RT SMT. The most notable applications include low-loss plasmonics^21^, smart window coatings^22^, ultrafast optical switches and sensors^11^, new electronic devices such as Mott field effect transistors^23^, and uncooled bolometers for infrared imaging^24^. Smart devices, which use the large optical and electrical property changes of VO_2_, appear to be at the forefront of this field and are of particular interest here. For example, a smart energy-efficient window must satisfy a number of criteria, including high transmittance in the visible range (400–700 nm), low transmittance in the infrared range (3–50 μm), and variable transmittance in the near-infrared range (700–3,000 nm). This variable transmittance depends on whether the building interior must be heated or cooled, i.e., the window transmits (reflects) the near-infrared light if there is a heating (cooling) demand^25^, and it must be actively or passively modulated. In this paper, we discuss a possible thin-film platform in which to electro-thermally control the VO_2_ phase across its SMT, which enables modulation for smart window thin-film coatings and/or other technological devices.

## Results

**Figure 1:**
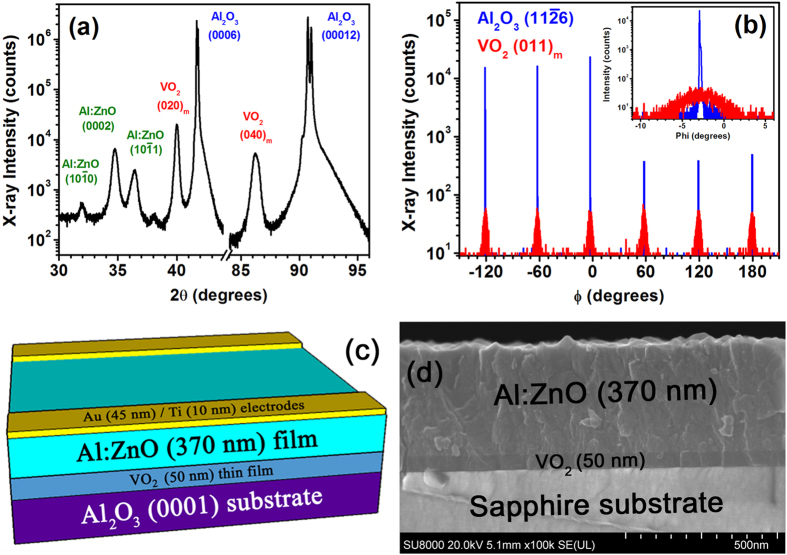
(**a**) Symmetric *θ-2θ* and (**b**) asymmetric *ϕ* scans of the multilayered thin-film samples revealing the epitaxial relationship between the film layers and substrate. (**c**) Schematic and (**d**) cross-sectional SEM image of the multilayered thin-film samples composed of Au/Ti electrodes on Al:ZnO (370 nm)/VO_2_ (50 nm)/Al_2_O_3_ (0001) substrate.

Epitaxial VO_2_ thin films were grown on c-plane (0001) sapphire (Al_2_O_3_) substrates using oxygen plasma-assisted pulsed laser deposition (PA-PLD). The SMT occurs near 50 °C, as confirmed with Raman spectroscopy and four-point probe electrical measurements, which are not shown here but can be found elsewhere in the literature^26^. The near-RT SMT of these VO_2_ thin films can be induced using the aforementioned techniques, most of which are external to the VO_2_ thin film and device. For example, the SMT is typically thermally induced using an external heater in contact with the VO_2_. However, the direct incorporation of a thin-film heater into a multilayered thin-film device has spatial and temporal advantages, low power requirements, and fast response times^27^,^28^. Therefore, a thick film of Al:ZnO, which has unique transparent heater qualities^29^, was grown on top of the VO_2_ thin films using atomic layer deposition (ALD). Details on these Al:ZnO films can be found in the literature^29^,^30^,^31^. The ALD-grown Al:ZnO film is polycrystalline and the VO_2_ thin film has an epitaxial relationship with the sapphire substrate of (010)[100]VO_2_ 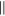 (0001)[10-10]Al_2_O_3_ as confirmed via symmetric [Fig. 1(a)] and asymmetric [Fig. 1(b)] x-ray diffraction (XRD) scans.

**Figure 2:**
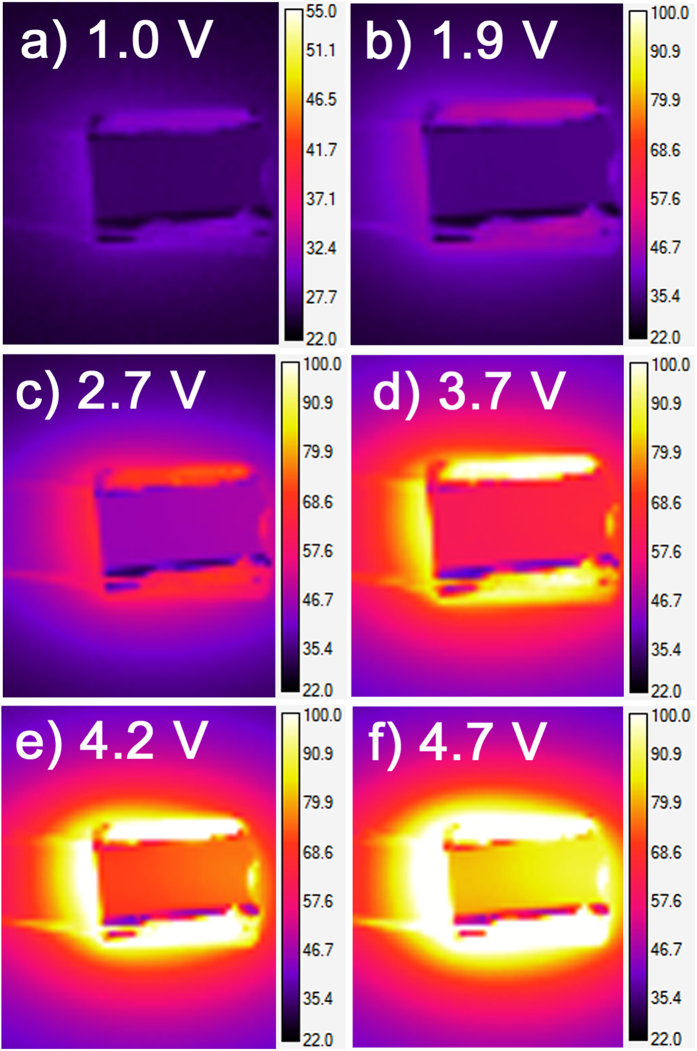
IR thermal images for the applied voltages of (**a**) 1.0 V, (**b**) 1.9 V, (**c**) 2.7 V, (**d**) 3.7 V, (**e**) 4.2 V, and (**f**) 4.7 V after the steady-state temperatures were reached.

**Figure 3:**
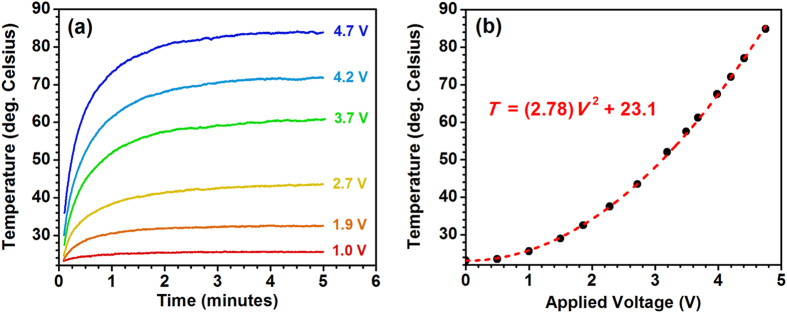
(**a**) Temperature versus time plots for the applied voltages in Fig. 2, where the steady-state final temperatures are achieved within 2–5 minutes. (**b**) Steady-state temperature versus the applied voltage, which shows the expected parabolic relationship between temperature and applied voltage. Measurements were taken with an IR thermal camera using the temperature at the center of the samples.

**Figure 4:**
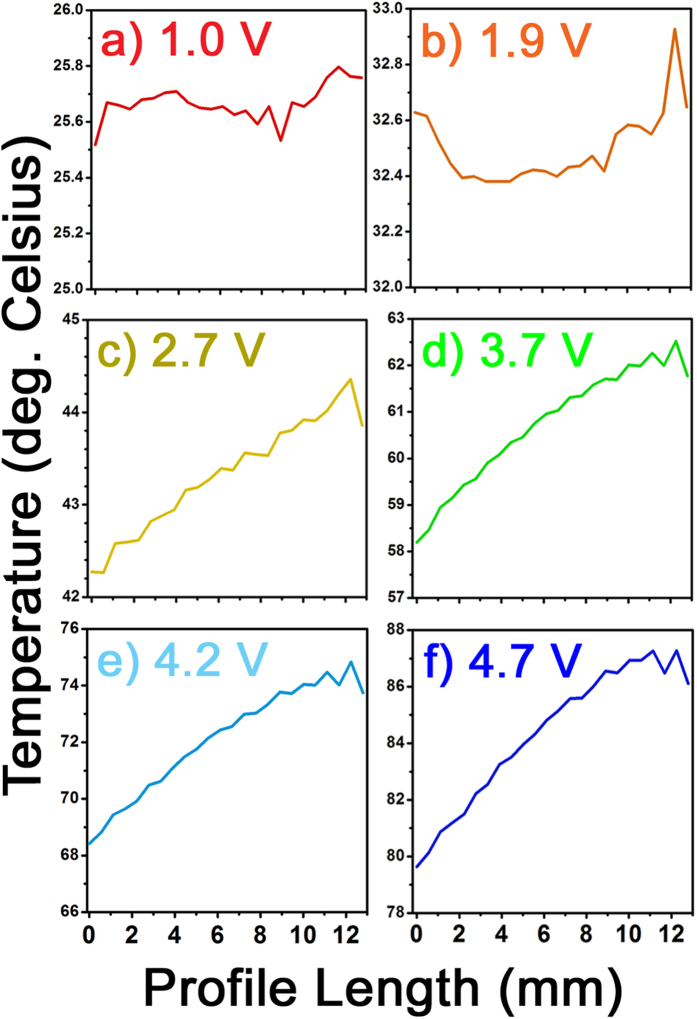
Temperature distribution across the sample images in Fig. 2, which are taken along the diagonal between the electrodes from the upper left to the lower right (12.8 mm in length). A fairly uniform temperature distribution (±1 °C below 3 V) is found for the applied voltages studied: (**a**) 1.0 V, (**b**) 1.9 V, (**c**) 2.7 V, (**d**) 3.7 V, (**e**) 4.2 V, and (**f**) 4.7 V.

Electrical contacts were fabricated on top of the Al:ZnO film along opposite edges of the 10 mm × 10 mm sample [see the multilayered thin-film sample schematic in Fig. 1(c)], such that the transparent heating properties of the Al:ZnO film could be used to induce the SMT in the underlying VO_2_ thin film. In addition to their transparency, the Al:ZnO films (with an Al:Zn ALD cycle ratio of 1:20) have a moderate thermal conductivity (4.2–4.3 W m^−1^ K^−1^), which facilitates heat transport to the VO_2_ thin film^29^. An infrared (IR) camera was used to measure the temperature distribution over the 1 cm^2^ area of the Al:ZnO/VO_2_ multilayered thin-film samples at different applied voltages, and these measurements were corroborated via thermocouple measurements. Figure 2 shows the steady-state IR thermal images of the multilayered thin-film samples at applied voltages of 1–5 V. These steady-state temperatures were achieved within 2–5 minutes after applying the voltages, where the time rates of temperature increase are shown in Fig. 3(a); the final steady-state temperatures are plotted versus the applied voltage in Fig. 3(b). The temperature was measured at the center of the samples, where there is a fairly uniform temperature distribution across the entire surface, particularly at or below the SMT. Figure 4 shows this temperature gradient across the multilayered thin-film samples, which was measured between the electrodes from the upper left to the lower right [see Figs 1(c) and 2]. The SMT in these multilayered thin-film samples occurs at just under 3 V (discussed later), where the temperature gradient is ±1 °C across the sample. Smaller temperature gradients are expected by improving the electrodes because of the noticeable defects in the upper and lower left corners of the sample (see Fig. 2), which cause the temperature to decrease at the 0 mm end (see Fig. 4).

The Al:ZnO film is heated because of the Joule heating effect, where the power converted to heat (and consequently the steady-state temperature) is proportional to the square of the applied voltage as shown in Fig. 3(b)^9^. This Joule heating effect can be reproducibly controlled using the Al:ZnO film properties, particularly the film thickness and amount of Al-doping (i.e., Al:Zn ALD cycle ratio), which govern the metallic behavior of the films based on the carrier concentration as we recently demonstrated^29^. These two parameters enable one to precisely tailor the amount of heat and temperature, where both thicker and more metallic-like films produce higher temperatures at lower applied voltages, an approach that has considerable advantages in device fabrication and operation.

**Figure 5:**
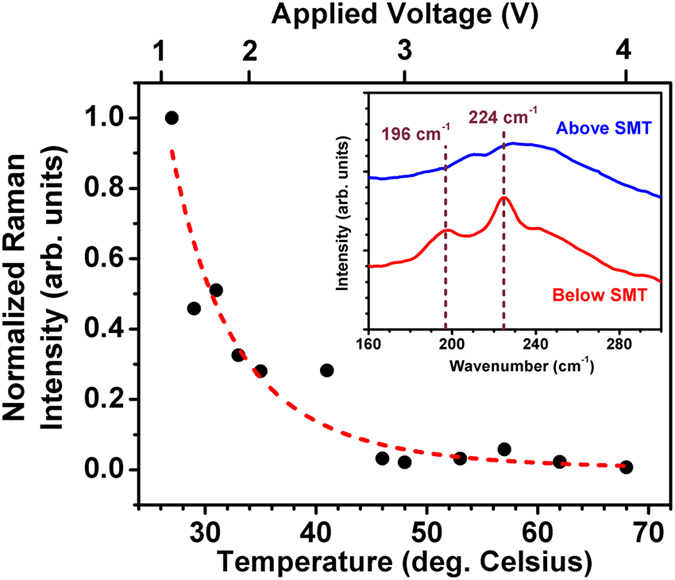
The 196 cm^−1^ and 224 cm^−1^ Raman modes of the VO_2_ monoclinic phase were tracked with respect to the temperature by applying voltage to the Al:ZnO/VO_2_ multilayered thin-film samples. An SMT temperature between 42 and 46 °C was found, where the red dashed line is a guide to the eye, which indicates a T^−5^ power law dependence. (Inset) Raman spectra of the multilayered thin-film samples below (red line) and above the SMT (blue line), which show that only the modes of the monoclinic semiconducting phase are present below the SMT.

The SMT of the VO_2_ thin film below the Al:ZnO film layer was tracked using various characterization techniques. Micro-Raman spectroscopy is suitable to distinguish the SMT in VO_2_. Below the transition, sharp Raman peaks (modes) are observed, which signifies the monoclinic semiconducting phase, whereas only a broadband emission is observed for the rutile metallic phase above the transition^26^,^32^. Many, but not all, of the possible Raman modes for the monoclinic semiconducting phase of VO_2_ were resolved in the RT spectra^26^,^32^,^33^,^34^,^35^. The inset of Fig. 5 shows the Raman spectra, which highlights the 196 and 224 cm^−1^ modes, for both VO_2_ phases: the semiconducting phase below the transition and the metallic phase above the transition. These two Raman modes were used to track the SMT in the VO_2_ thin film to determine the transition temperature of the Al:ZnO/VO_2_ device, which was found to be between 42 and 46 °C (see Fig. 5). Similar and corroborating results were obtained using an external heater below the sapphire substrate to increase the temperature of the VO_2_ thin film (not shown).

**Figure 6:**
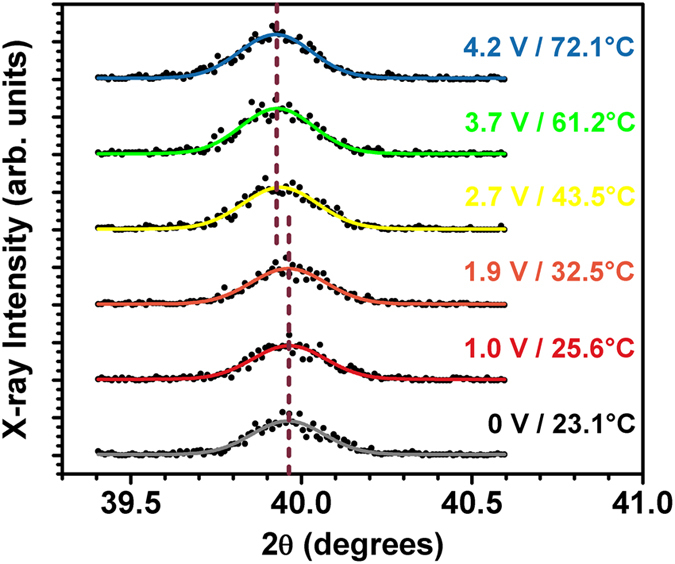
Symmetric *θ-2θ* XRD scans of the Al:ZnO/VO_2_ multilayered thin-film device at various applied voltages/temperatures. The VO_2_ (020)_m_ reflection suddenly shifts to a lower angle after the SMT is crossed at 43.5 °C.

XRD was also used to track the SMT in the VO_2_ thin film as shown in Fig. 6 when voltages were applied to the Al:ZnO/VO_2_ multilayered thin-film device. The XRD results show that there is a sudden shift in the VO_2_ unit cell parameters at an SMT temperature of 43.5 °C, thus corroborating the Raman spectroscopy results. Below this temperature, the monoclinic VO_2_ (020) reflection is centered at *2θ* = 39.962° and yields a lattice parameter of 0.2254 nm. The VO_2_ reflection suddenly shifts to a lower angle (*2θ* = 39.924°) above this temperature and yields an expanded lattice parameter of 0.2256 nm. These results are consistent with the bulk VO_2_ x-ray powder diffraction files (PDFs) for the monoclinic (01-082-0661) and rutile (03-065-9786) phases from the International Centre for Diffraction Data (ICDD) and published XRD studies in the literature^36^. The shift in the bulk VO_2_ structures amounts to ∆*2θ* = 0.09°, whereas only a shift of ∆*2θ* = 0.04° is observed in the multilayered thin-film device. This result is not surprising because the VO_2_ thin film is under stress due to the mismatch with the sapphire substrate and the Al:ZnO film on top of it. Furthermore, thermal expansion of the VO_2_ lattice cannot account for this shift. First, the peak shift is sudden and not gradual with increasing temperature. Second, the average thermal expansion of VO_2_ is *α*_*ave*_ = 5.70 × 10^−6^ K^−1^ (monoclinic structure) and 13.35 × 10^−6^ K^−1^ (rutile structure) at these temperatures^37^, which can only account for approximately 10% of the exhibited lattice expansion (i.e., *α*_*ave*_ must be an order of magnitude larger to be responsible for the observed XRD peak shift in the data).

**Figure 7:**
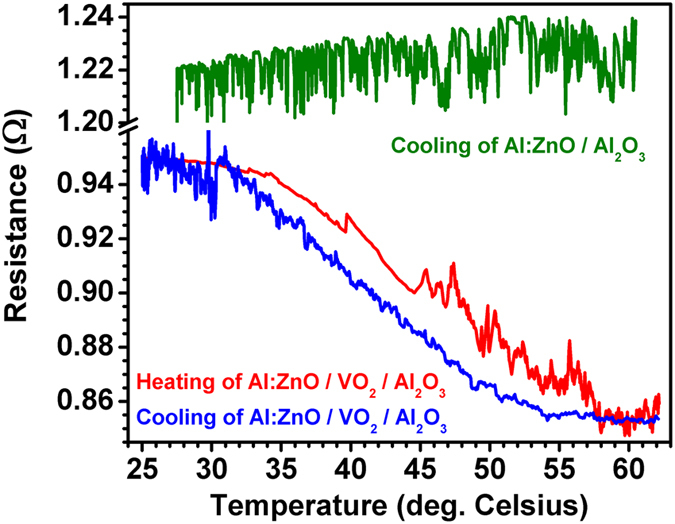
Resistance versus temperature plots obtained from four-point probe electrical (I–V) measurements in the van der Pauw configuration. The Al:ZnO/VO_2_ multilayered thin-film device shows the expected hysteresis of the VO_2_ SMT during heating (red) and cooling (blue). Measurements for an identical Al:ZnO film on sapphire without the VO_2_ thin film shows no hysteresis (green).

Electrical transport measurements in the form of electrical resistance versus temperature plots are typically used to study the SMT of VO_2_ films. In this case, the VO_2_ thin film in the Al:ZnO/VO_2_ multilayered thin-film device is buried beneath the Al:ZnO film and therefore cannot be measured directly. However, the electrical resistance of the Al:ZnO film can be measured versus temperature using a four-point probe in the van der Pauw configuration and a resistive heater below the sapphire substrate. Figure 7 shows that indeed the SMT of the underlying VO_2_ thin film is manifest in the measurements of the Al:ZnO resistance, with the expected hysteresis during heating and cooling. Furthermore, an identical Al:ZnO film without the underlying VO_2_ thin film was measured for comparison and shows no hysteretic behavior.

Interestingly, the growth of an Al:ZnO film on top of the VO_2_ thin film provides several advantages in addition to placing a thin-film heater in direct contact with the VO_2_ (previously mentioned), which creates a mechanism to finely control the heat applied to VO_2_ to induce the SMT. The thick Al:ZnO film also acts as a capping layer to the VO_2_ thin film and prevents the oxidation of VO_2_ to V_2_O_5_, which is the most energetically favorable and stable phase. Otherwise, over the lifetime of a device, the VO_2_ thin film will oxidize to become V_2_O_5_ and degrade the SMT and its associated properties. Another advantage of the thick Al:ZnO film is its ability to decrease the SMT of the VO_2_ thin film by approximately 5–10 °C compared to a VO_2_ thin film without an Al:ZnO capping film^26^. This phenomenon is thought to occur because of the additional stress on the VO_2_ thin film and/or an alteration of the oxygen vacancy concentration, which pushes the SMT to a lower temperature^38^.

In summary, the electro-thermal control of a multilayered Al:ZnO/VO_2_ thin-film device was demonstrated, where the SMT of the VO_2_ thin film was induced by applying a small potential (<3 V) across the Al:ZnO film. This electro-thermal energy provided by the Al:ZnO film can be finely tuned using its transparent conducting oxide properties to significantly control the VO_2_ SMT and its associated electrical and optical properties. The Al:ZnO film acts as a transparent window and heater, serves as a protective capping layer to the VO_2_ thin film, and aids in decreasing the VO_2_ transition temperature. These results have important implications for the use of VO_2_ and Al:ZnO in technological applications, particularly active smart devices.

## Methods

### Fabrication of the samples

Epitaxial vanadium dioxide (VO_2_) thin films were grown on non-annealed c-plane (0001) sapphire (Al_2_O_3_) substrates (10 mm × 10 mm) at 550 °C using a Neocera pulsed laser deposition (PLD) system, which operated at a base pressure of 10^−8^ Torr. The V_2_O_5_ target material was ablated with a KrF excimer laser (λ = 248 nm, pulse width = 25 ns, energy = 220 mJ/pulse) at an angle of 45° with a repetition rate of 3 Hz and a spot size of approximately 2 mm × 4 mm, which resulted in an energy density of ~3 J cm^−2^. These 50 nm thick VO_2_ thin films, confirmed with x-ray reflectivity and cross-sectional scanning electron microscopy (SEM) [Fig. 1(d)], were grown at a rate of ~0.4 nm/min under a 150 W oxygen (O_2_) radio frequency plasma using ultra-high purity (99.994%) O_2_ gas at a working pressure of 3.0 × 10^−5^ Torr (i.e., plasma-assisted PLD). Further details on these VO_2_ thin films are discussed elsewhere^26^.

Aluminum-doped zinc oxide (Al:ZnO) thin films were grown on top of the epitaxial VO_2_ (020)_m_ thin films using a Cambridge NanoTech Ultratech atomic layer deposition (ALD) system, which operated at a base pressure in the mid-10^−3^ Torr range. Ultra-high purity (99.999%) nitrogen (N_2_) gas, which constantly flowed at 20 sccm, was used to purge the chamber and as the carrier gas for the precursors: diethyl zinc [DEZ, Zn(C_2_H_5_)_2_], water (H_2_O), and trimethylaluminum [TMA, Al(CH_3_)_3_]. The Al:ZnO films were grown at 250 °C by alternating between 15 ms pulses of DEZ and H_2_O (with 5 s purges in between each) for 20 times (cycles) to obtain a zinc oxide film. After the 20^th^ DEZ pulse, a 15 ms pulse of TMA was used for the aluminum dopant, which was followed by the 20^th^ H_2_O pulse. This 20 cycle sequence defines a 1:20 Al:Zn ratio and consequently constitutes the Al:ZnO film used herein. In total, 3000 cycles were performed, which yielded a growth rate of ~0.12 nm per cycle and thus a total thickness of 370 nm [Fig. 1(d)]. Further details on these ALD pulsing sequences and Al:ZnO thin films are discussed elsewhere^30^,^31^.

### Fabrication of the electrical contacts

45 nm gold (Au)/10 nm titanium (Ti) electrical contacts (1 mm × 10 mm) were deposited on top of the Al:ZnO/VO_2_ multilayered thin film on opposing sides of the 10 mm × 10 mm sample [Fig. 1(c)]. These contacts were deposited at RT with a growth rate of ~0.12 nm/sec in an AJA International electron beam evaporation system, which operated at a base pressure of 10^−8^ Torr.

### SEM imaging techniques

A Hitachi SU8010 field-emission scanning electron microscope (FE-SEM) was used to obtain cross-sectional images of the multilayered Al:ZnO/VO_2_ thin-film device after cleaving it in half.

### Raman spectroscopy characterization techniques

A Horiba LabRAM HR Evolution Raman spectrometer with a laser excitation wavelength (λ = 785 nm) in the near-infrared range was used because it has been shown to produce the clearest spectrum of Raman active modes for VO_2_ on sapphire compared to shorter wavelengths in the visible range^35^. This clearer spectrum is achieved because resonance fluorescence at these shorter visible wavelengths increases the background and overwhelms the signal from the VO_2_ thin film. The spectra were acquired for the Al:ZnO/VO_2_ thin-film device at various steady-state temperatures by applying a voltage across the electrical contacts on the Al:ZnO film and using an external resistive heater below the sapphire substrate to increase the temperature.

### XRD characterization techniques

A standard high-resolution (0.0001°) four-circle x-ray diffractometer (XRD) with Cu K_*α*_ radiation and thin-film optics was used to characterize the microstructure of the Al:ZnO/VO_2_ thin-film device. Diffraction scans were acquired for the Al:ZnO/VO_2_ thin-film device at various steady-state temperatures by applying a voltage across the electrical contacts on the Al:ZnO film and using an external resistive heater below the sapphire substrate to increase the temperature.

### Electrical transport characterization techniques

The temperature dependence of the electrical resistance of the multilayered Al:ZnO/VO_2_ thin-film device was measured using a four-point probe in the van der Pauw configuration, where the SMT of the VO_2_ was thermally induced with a resistive heater below the sapphire substrate. The four, 50 μm diameter probes contacted the Al:ZnO film surface, where current-voltage (I–V) measurements were acquired versus temperature using a Keithley 6220 Current Source applying 21 mA of direct current along one edge of the sample and a Keithley 2182A Nanovoltmeter measuring the potential across the other edge.

## Additional Information

**How to cite this article**: Skuza, J. R. *et al.* Electro-thermal control of aluminum-doped zinc oxide/vanadium dioxide multilayered thin films for smart-device applications. *Sci. Rep.* **6**, 21040; doi: 10.1038/srep21040 (2016).
